# Supplementary material for: Mechanisms governing the pioneering and redistribution capabilities of the non-classical pioneer PU.1
Source: Nat Commun. 2020 Jan 21;11:402. doi: 10.1038/s41467-019-13960-2 (PMC6972792; doi:10.1038/s41467-019-13960-2)
Supplement: Supplementary file 7 — Source data [file 41467_2019_13960_MOESM7_ESM.zip › Source_Data/FigureS2/FigureS2B_MotifScanOutput/KmeansCluster3/homerResults/motif33.similar.html]

motif33

## Information for motif33

A
C
T
G
A
G
T
C
C
G
T
A
A
C
T
G
C
T
A
G
G
T
C
A
A
C
T
G
C
G
T
A
C
T
A
G
C
G
T
A
A
T
C
G
C
G
T
A
A
C
T
G
  
Reverse Opposite:  

G
T
A
C
G
C
A
T
A
T
G
C
C
G
A
T
A
G
T
C
C
G
A
T
T
A
G
C
C
A
G
T
G
A
T
C
A
G
T
C
G
C
A
T
C
T
A
G
A
G
T
C
  

|  |  |
| --- | --- |
| p-value: | 1e-28 |
| log p-value: | -6.528e+01 |
| Information Content per bp: | 1.894 |
| Number of Target Sequences with motif | 52.0 |
| Percentage of Target Sequences with motif | 1.09% |
| Number of Background Sequences with motif | 37.6 |
| Percentage of Background Sequences with motif | 0.09% |
| Average Position of motif in Targets | 92.5 +/- 39.8bp |
| Average Position of motif in Background | 112.6 +/- 72.2bp |
| Strand Bias (log2 ratio + to - strand density) | 0.0 |
| Multiplicity (# of sites on avg that occur together) | 1.00 |
| Motif File: | file (matrix) reverse opposite |

### Similar de novo motifs found

|  |  |  |  |  |  |  |  |
| --- | --- | --- | --- | --- | --- | --- | --- |
| Rank | Match Score | Redundant Motif | P-value | log P-value | % of Targets | % of Background | Motif file |
| 1 | 0.974 | T C G A C T A G A T G C C G T A C T A G T A C G G T C A A C T G C G T A T C A G C G T A A C T G C G T A C A T G | 1e-21 | -50.270495 | 0.96% | 0.11% | motif file (matrix) |
| 2 | 0.838 | A C G T A G T C A C G T A G T C A G C T A G T C A T G C C G A T A C T G A G T C A G C T T C G A | 1e-19 | -45.755158 | 1.01% | 0.14% | motif file (matrix) |
| 3 | 0.918 | A C T G A G T C C G T A T C A G C T A G G T C A A C T G C G T A A C T G C G T A A T C G | 1e-16 | -37.091793 | 1.45% | 0.39% | motif file (matrix) |
